# *TactCube*: An Intelligent Device to ‘converse’ with Smart Environments

**DOI:** 10.3390/s22145235

**Published:** 2022-07-13

**Authors:** Pietro Battistoni, Marianna Di Gregorio, Marco Romano, Monica Sebillo, Giuliana Vitiello

**Affiliations:** Computer Science Department, University of Salerno, Via Giovanni Paolo II, 132, 84084 Fisciano, Italy; pbattistoni@unisa.it (P.B.); madigregorio@unisa.it (M.D.G.); marromano@unisa.it (M.R.); gvitiello@unisa.it (G.V.)

**Keywords:** conversational AI, human–computer interaction, ambient intelligence, wireless device, tangible interface, tactile interface, haptic interface, ESP32

## Abstract

Ambient Intelligence is a vision of daily life in which intelligent devices interact with humans to make their lives easier, and the technology is invisible. Artificial Intelligence (AI) governs this smart environment and must interact with humans to best meet their needs and demands. Although voice assistants are very popular and efficient as conversational AI, under some conditions they cannot be used. Therefore, this work proposed a complementary tactile and tangible interface to converse with AI, creating new *Tactile Signs*. A prototype of *TactCube*, a wireless cube-shaped device that can interact with AI using only the tactile sense, is presented. The hypothesis is that *TactCube* can be manipulated with one hand and generate a sequence of numbers that can be interpreted as a new tactile language by a neural network solution. The paper describes the initial research made to define how these sequences can be generated and demonstrates how *TactCube* is able to do it.

## 1. Introduction

Ambient Intelligence (AmI) as a term was coined in 1998 by Eli Zelkha with Simon Birrell [1]. It is a vision of daily life where intelligent devices interact with humans to make their lives easier, and the technology is invisible. The advances made on hardware and software for Artificial Intelligence (AI) solutions will encourage the AmI paradigm even more than in the past. To comply with this paradigm, the interaction between humans and the AI should be as natural as possible. Thus, speech-enabled applications have become popular, offering human-like interactions between computers and users, while hiding all the technology behind. Representative examples of these conversational interfaces are, Google Home^®^, Amazon Alexa^®^ and Apple Siri^®^.

Based on conversational AI [2] methodologies, these solutions have obvious advantages as well as some obvious limitations.

The most significant limitations are:

- Vocal assistants do not include people with a language-impaired capacity or deafness in the conversation;
- Vocal assistants are challenging to use in a noisy or crowded environment;
- Voice assistants disturb when used in quiet environments, such as libraries and study rooms.

It is worth noting that even a perfect conversational AI will suffer the same limitation of conversation among humans: misunderstandings, difficulties in interpreting different languages, dialects and idioms. If the user suffers from some communication disorder or disability, these issues become even more severe.

At the CHI 2016 conference on Human Factors in Information Systems, a position paper on multisensory interaction was presented [3] which evaluated alternative senses to audio and visual. Taste and smell began to be evaluated as possible additional means of interaction with computers, but in the conclusions, the authors predicted that *touch* would be increasingly exploited.

The impact of touching behavior on daily life is sufficiently investigated in social, medical, and psychological research [4,5,6], emphasizing that *touch* is commonly used as a non-visual and non-verbal form of communication.

At the LabGIS laboratory in the department of computer science at the University of Salerno, new human–computer interaction (HCI) modalities are investigated to improve the conversational AI skill to better contextualize users’ requests.

The goal of the research described in this paper concerns the design and implementation of a tangible user interface that will be used in future work to investigate *touch* as an additional means of interaction, proposing a device designed with both user comfort and the need for additional channels of interaction with AI in mind. It describes the design, development and initial test of a preliminary prototype of the interactive cube = shaped device, namely *TactCube*.

Like most TUIs, *TactCube* is one of the sensing-based interaction devices that brings with it the typical challenges of its kind [7].

- The sensing-based interaction must be useful and active, but not disturb the user.
- The system must know that the user is addressing it and not other systems.
- The user must know that the system is responding to his request.
- The system must know which object the user’s command refers to.

The *TactCube* was designed to solve these challenges, and because its technical characteristics are critical to success, they are well detailed in the paper.

The following section shows some related works. Section 3 describes the rationale underlining the *TactCube* design. Section 4 contains a description of how it was realized and reports the principles of its operations. Section 5 explains the methods used for experimentation, while Section 6 contains a brief discussion of results and how they can be interpreted. A final section reports conclusions and outlines future work.

## 2. Related Work

The goal of this Section is to summarize the most relevant research work examined to study the basic principles of the tactile interfaces and the progress achieved in their realization.

In [8], Ishii et al. established a new type of HCI, named the “Tangible User Interface” (TUI). TUI, grounded on the previous work by Fitzmaurice et al. [9] about a graspable user interface, intended to join the physical world in HCI moving beyond the graphic user interface (GUI). At that time, a GUI was represented by the desktop metaphor simulating a physical desktop on a bit-mapped screen [10]. Today, the pervasiveness of touch screens have brought forward the GUI and the TUI, as initially conceived, probably becoming farther from their original goal.

Those works are not strictly related to the recommended *TactCube* device, which instead intends to offer a tool to converse with AI, avoiding visual information display.

X. Liu et al. [11] propose a mobile shape-changing peripheral that enables a text-based AI conversational agent to deliver physical interactions in real time during mobile chat conversations. The authors’ goal was to increased levels of empathy for the AI agent. Although this proposal represent an example of TUI applied to AI interaction, it requires the use of an additional visual interface for the text conversation, which *TactCube* aims to avoid. A recent article [12] presents a broad overview of TUI in many areas, focusing on its technical solutions. None of the works reported aims to be an interface for conversing with artificial intelligence, and furthermore, none of them exclusively uses the tactile sense, which is a primary feature of the proposed *TactCube*.

The *TactCube* would be a non-verbal and non-visual conversational tool adopting the tactile sense only. In [13], the authors propose a haptic wheel, a freestanding single-axis rotational controller incorporating a vibro-tactile cue prototype device for tangible HCI. The *haptic wheel* is cost-effective according to the authors and combines haptic with tangible interaction as does the *TactCube*. However, the *haptic wheel* offers fewer conversational options with a single rotation axis and is designed to stay on a tabletop, while the *TactCube* offers multiple axis rotations and is designed to be mobile and stay in a pocket, as better detailed in next section.

## 3. The *TactCube* Concepts

What is expected from the AmI is that it perceives users’ needs, acquires their explicit requests and makes decisions on how to govern the environment to satisfy all these requirements.

The AmI can count on a wide range of local and remote sensors to perceive the current environment and acquire information and news from the network to infer some well-contextualized decisions. These tasks can probably be performed better than by humans thanks to the availability of senses that the AmI can have and the speed with which it can acquire information. However, to complete the contextualization, the AmI needs to know users’ feelings and desires when the decision has to be made, and to achieve this, it needs to converse with them.

Inspired by the previous research about the sign languages interpretation through AI, Refs. [14,15,16], the proposed *TactCube* device would be used in future work to create manipulation-generated *tactile signs* that could be interpreted by AI.

A previous study on guessability was presented at the INTERACT 2021 conference. It led to insights used to design interfaces that are truly meaningful to users interacting with the AmI [17]. Since further work is needed from the users’ perspective, the present prototype was also built to conduct such experiments.

By design, the device should be small enough to stay in a pocket and be utilized by one hand only. To be inexpensive and affordable to produce, it should be made with off-the-shelf components. Eventually, the device should have a cube shape and be manipulated by one hand only.

As possible manipulations, the hand holding the device can perform rotations on the three axes and a tap sequence on the face under the index finger.

**Figure 1:**
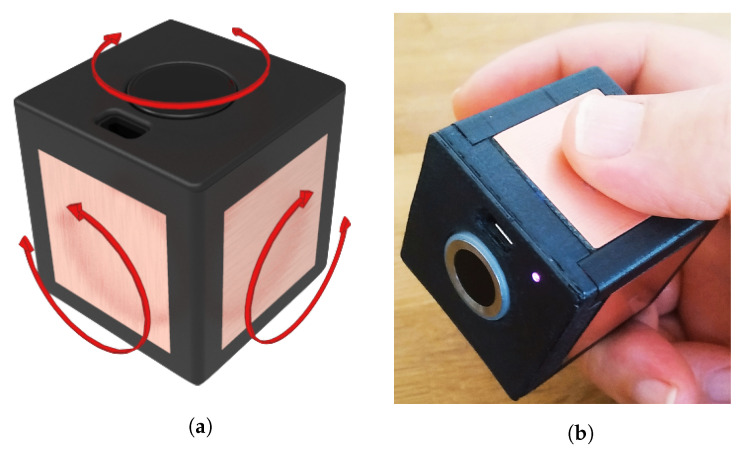
The possible *TactCube* rotations: (**a**) rotations axes; (**b**) hand grasping.

Figure 1a gives an example of possible rotations.

As the hand rotates the *TactCube*, it will touch different faces of the cube in turn. Together with the final tap sequence, the rotations sequence will represent the message for the AmI.

## 4. The *TactCube* Device

This section describes the hardware design, the components used to build the prototype of the *TactCube*, and the software used to test the device and investigate its further use.

**Figure 2:**
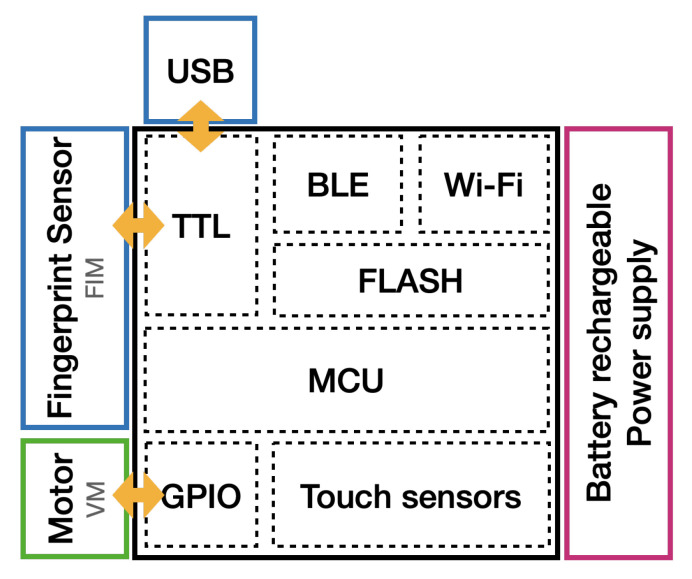
Functional modules of the prototype.

Figure 2 shows the functional modules of the electronic prototype for the *TactCube*. Its main component is the ESP32 System on Module (SoM) [18] with a generic microcontroller unit (MCU), 4 MB of flash memory for data and program, two CPU cores that can be individually controlled, and a rich set of peripherals, including capacitive touch sensors.

The ESP32 requires a power supply of 3.3 V, given by a small external circuit that converts a rechargeable lithium polymer battery (LiPo) voltage of 3.7 V to a stable 3.3 V. An additional constant voltage and constant current charging circuit takes the 5 V from the USB port and recharges the small 400 mAh LiPo battery. The USB port is also utilized for the firmware upload and as a program debug port.

The fingerprint identification module (FIM) can autonomously collect fingerprint images and perform fingerprint enrollment, identification and verification functions. The FIM is connected to the MCU by the transistor–transistor logic (TTL) serial interface circuit. The MCU sends instructions to the FIM, which returns a reply package. A vibrating motor (VM) is connected to one general purpose input output (GPIO) port to reproduce a sequence of haptic feedback. Finally, five capacitive touch sensors detect when a finger touches the respective face of the cube.

**Figure 3:**
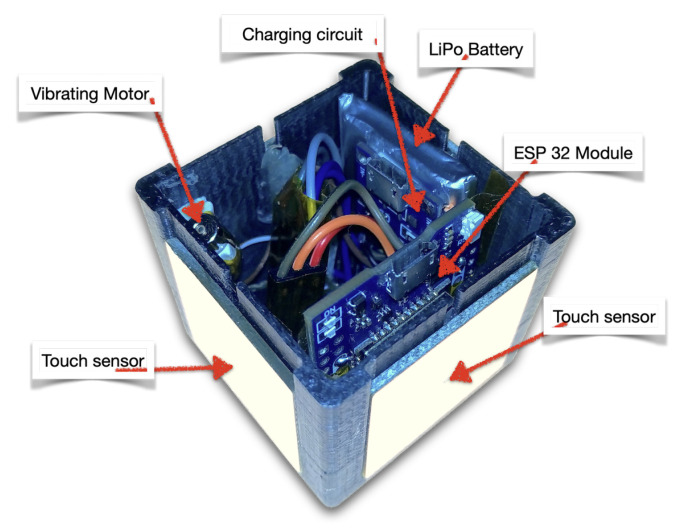
Internal view of the prototype.

Figure 3 shows an internal picture of the prototype.

**Figure 4:**
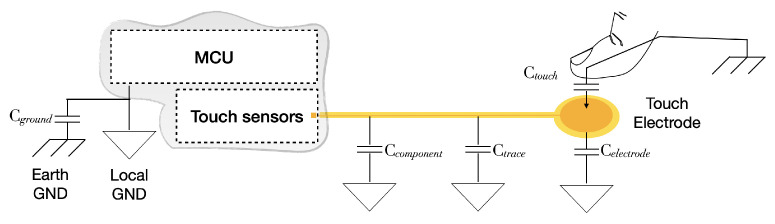
Touch sensor system.

Figure 4 shows the touch sensor circuit which measures the total capacitance on the touch channel. When the capacitance changes and the magnitude of the change exceeds the threshold value, the system detects the “finger touch”.

When the touch electrode is not in contact with a finger, the capacitance *C* of the circuit is given by the combination of all the parameters $C_{ground},\;C_{component},\;C_{trace},\;C_{electrode}$ (Table 1).

When a finger touches one or more of the touch electrodes, $C_{touch}$ is the total change ΔC in capacitance which triggers the “finger touch” event. A calibration phase at power on will measure the *C* and use it to determine the ΔC when one or more faces are touched. Since the touch sensor can be negatively affected by the surrounding environment, the calibration phase smooths out any environmental fluctuation that might affect the sensor response under varying conditions.

**Figure 5:**
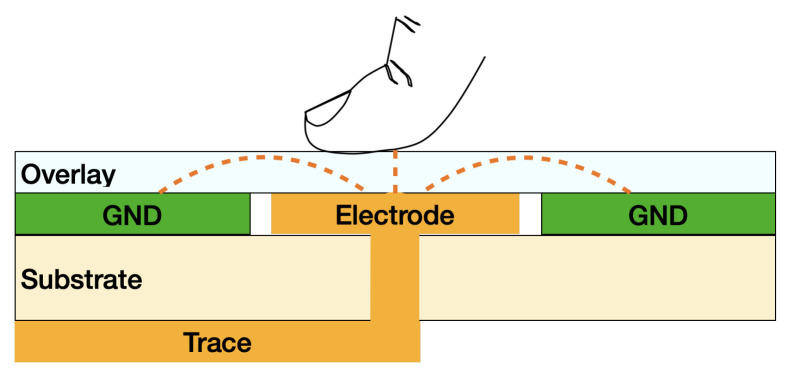
Schematic structure of a touchpad.

Figure 5 shows the schematic structure of an ideal touchpad. The finger and the copper pad represent the conductive parallel plate of a capacitor separated by a dielectric medium, the overlay.

To optimize the response of the sensor, it is necessary to decrease the *C* at the design stage by reducing the length of the traces and optimizing the PCB layout and, at the same time, to increase the $C_{touch}$ as much as possible. $C_{touch}=\frac{\epsilon \times S}{4\times \pi \times k\times d}$, where ϵ is the dielectric constant of the overlay, *S* is the area that the finger touches on the electrode, *k* is the electrostatic constant, and *d* is the thickness of the overlay. Thus, eliminating air gaps between the electrode and the overlay, using high dielectric constant materials as overlay, reducing the thickness of overlay, and enlarging the electrode area as much as possible, will increase $C_{touch}$ as desirable. In addition, an appropriate trigger threshold is needed to avoid unintentional touch and ensure strong sensitivity even under varying conditions of finger contact, such as a wet hand or dry air, etc.

Two strategies were used for the prototype of touch pads. Initially, five square printed circuit boards were placed on each of the five faces of the cube with an FR4 fiberglass substrate and a single side copper layer. The last prototype, on the other hand, used an adhesive copper sheet glued directly onto the polylactide (PLA) faces of the cube. Both solutions used a conformal silicone as a coating, which is a very thin dielectric medium. The two solutions did not show any appreciable difference on tactile performance, but the second solution allowed for a reduction in the size of the cube, decreasing the thickness of each face by about 1.5 mm.

**Figure 6:**
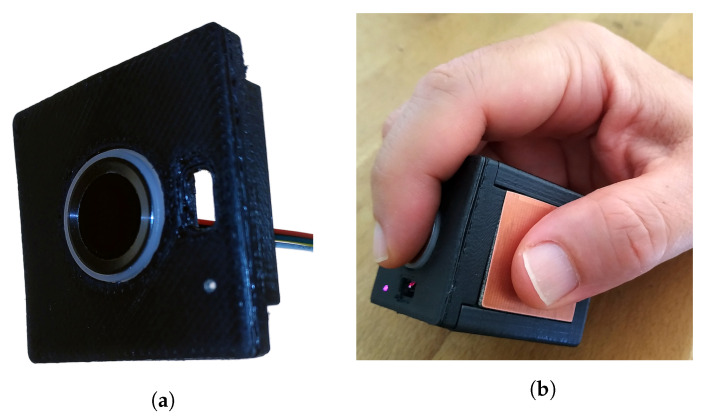
Fingerprint sensor: (**a**) fingerprint sensor module; (**b**) starting position.

FIM is intended to recognize users and apply their personal profiles (Figure 6a) so that multiple users can utilize the same *TactCube*, customizing its behavior. FIM works in conjunction with a host, which in this case is the *TactCube* MCU.

The user’s registration and identification are performed by these two simple commands:

i AutoEnroll. Once the MCU sends an *AutoEnroll* command, the FIM waits for acquiring fingerprint twice, extracts the features, and generates the registration template to store in its internal database. The FIM can manage up to 200 different users.
ii AutoIdentify. To identify the user, the MCU sends the “AutoIdentify” command to the FIM, which will answer with fingerprint ID matched, or “No matching fingerprints” otherwise.

Since the FIM firmware is proprietary and embedded in the commercial module, no tests are reported about it in this work. Furthermore, it should be noted that the FIM feature is optional and only oriented to make the device not personal, which is of minor importance for the scope of this research.

However, the fingerprint feature has an essential role in waking the device from a deep sleep and in starting the manipulation from a prominent position, as explained below.

Indeed, when not used, the device goes in a deep sleep mode to achieve long-lasting battery power. Only the ultra-low-power (ULP) coprocessor remains powered during this condition, maintaining a minimal power consumption [19]. Since the FMI sends a signal to the ULP when a finger is detected on the fingerprint sensor, a shortcode is programmed to awake the central processors when such a signal is received. Hence, the user is instructed to grasp the cube holding the index finger on the sensor to activate the device. This action gives the initial position from which every successive sequence starts (Figure 6b).

**Figure 7:**
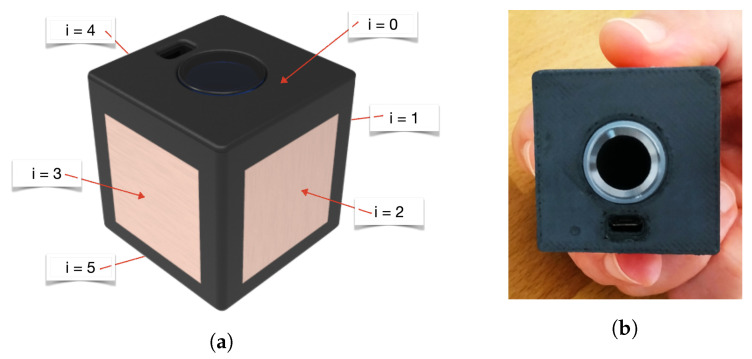
*TactCube* faces: (**a**) Numbered faces; (**b**) touched faces.

The main idea is that by rotating the device, users’ fingers touch the faces of the cube in sequence. The faces touched at each step of the rotation are detected by capacitive touch sensors placed on the cube. The device has six faces, one is used for FIM, the remaining for five touch sensors. Each touch sensor is connected to one of five inputs of the ESP32 touch sensor interface and numbered with 1≤i≤5 (Figure 7a).

We define the following function *f*:

(1) $$f\left(i,t\right)=\left\{\begin{array}{cc} 0 & \mathrm{if}\;\mathrm{the}\;i\mathrm{th}\;\mathrm{face}\;\mathrm{is}\;\mathrm{untouched}\;\mathrm{at}\;\mathrm{time}\;t\\ 1 & \mathrm{if}\;\mathrm{the}\;i\mathrm{th}\;\mathrm{face}\;\mathrm{is}\;\mathrm{touched}\;\mathrm{at}\;\mathrm{time}\;t \end{array}\right.$$

and assign to each touch sensor input *i* the value $\left\{s_{i}=2^{i}|1\leq i\leq 5\right\}$. The state *S* of touch sensors at time *t* is calculated as:

(2) $$S\left(t\right)=\sum_{i=1}^{5}s_{i}*f\left(i,t\right)$$

According to Equation (2), when the user holds the *TactCube* at time *t* (Figure 7b), the state of touch sensors will be calculating as $S\left(t\right)=2^{4}+2^{1}+2^{2}=22$. The value 22 is generated only when the faces i=4,i=1,i=2 are simultaneously touched, and every combination of the five touch sensors generates a unique decimal value between 2 and 62.

While manipulating the *TactCube*, the user will generate a sequence

(3) $$X=\{S\left(t_{0}\right),S\left(t_{1}\right)...S\left(t_{\left(n-1\right)}\right)\}$$

where $t_{0}$ is the time when the user started the manipulation, and $t_{n-1}$ is the time when the user ended the manipulation. This sequence can be seen as somewhat similar to a static sign. The beginning and end of a sequence are detected when either of the following two criteria is met:

(1) The user does not manipulate the device for a time longer or equal to σ, where σ is a constant of time lap to be established by experiments. Thus, $(t_{n}-t_{n-1})\geq \sigma$, having $t_{n-1}$ as the end of sequence and $t_{n}$ as the start of a new one.
(2) The user stops a rotation and performs a sequence of taps on the *TactCube* face under the index finger.

Since $t_{j+1}-t_{j}$ is the time interval that the user takes to change the *TactCube* position from position *j* to position j+1, and given $t_{0}$ as time of start of the *X* sequence and $t_{n-1}$ the time at end of the *X* sequence, we have that:

(4) $$\forall X\exists R_{X}=\overset{n-2}{\underset{j=0}{⋃}}\left(t_{j+1}-t_{j}\right)$$

The sequence $R_{X}$ can be defined as the *rhythm* of *X*. As expressed by Equation (4), the same *X* can have various *rhythms*.

Then

(5) $$M=\{m|m=\left(X,R_{x}\right)\}$$

is the set of all distinct *X* sequences generated by manipulating the *TactCube* with a specific *rhythm*.

**Figure 8:**
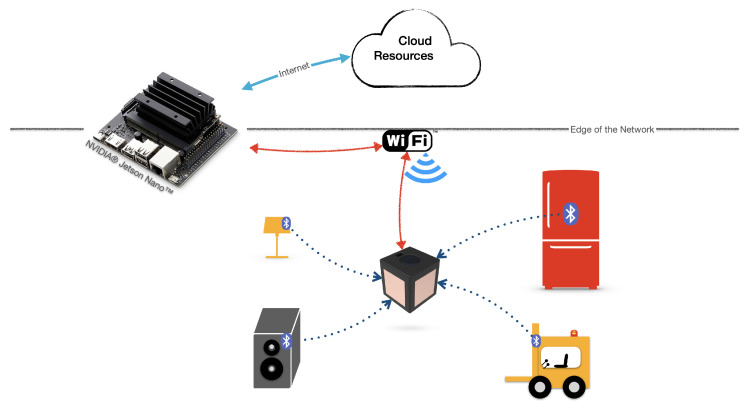
Schema of the proposed architecture.

The *TactCube* communicates with the AmI by Wi-Fi connectivity and sends the AmI the manipulation sequences and the proximity data about other BLE devices. The latter allow the AmI to achieve a more precise user’s location. Indeed, using the principles of “inquiry” and “inquiry scan”, as specified in the Bluetooth core specification [20], a BLE client application can discover services offered by other BLE server devices in its proximity. In the proposed case, the *TactCube* is programmed to be a BLE client that, discovering the devices in proximity, identifies them and estimates which is the nearest by evaluating the received signal strength indicator (RSSI) of each of them. Figure 8 depicts the architecture, where the BLE protocol detects the devices in proximity of the *TactCube*, and the Wi-Fi connects the *TactCube* with the AI of the AmI.

The ESP32 SoM integrates Bluetooth*®*, Bluetooth low energy (BLE) and Wi-Fi wireless connectivity options. The BLE offers wireless connectivity for compact battery-powered applications. It is highly efficient, minimizing the energy required to transfer data.

The AmI can send commands to the *TactCube* that actuate sequences of vibrating feedbacks or setup some of process variables such as, for example, the touch sensor sensitivity. All communications between the AmI and *TactCube* are performed by the MQTT protocol.

MQTT is designed as an extremely lightweight *publish/subscribe* messaging transport. It is an OASIS Standard [21] providing a reliable *client/server* messaging transport protocol suitable for communication in M2M/IoT contexts wherever a small code footprint is required, such as for the proposed *TactCube* hardware. MQTT supports two-way messaging, deterministic message delivery, basic QoS levels, always/almost connected scenarios, loose coupling and scalability to accommodate large numbers of devices as well. Based on the *client/server* paradigm, the MQTT server side is an application that acts as broker among the clients that can publish and subscribe to specific *topics* or a group of them. Each message is associated to a *topic* (sometimes defined as a *channel*), and the MQTT broker routes the published messages to all the clients that subscribed to its topic. Whenever a client publishes a message on a *topic*, all clients subscribed to that *topic* will receive it in an asynchronous way as soon the connection with the *broker* is available. As transport protocol, the MQTT protocol uses the Internet protocols (IP), such as the transmission control protocol (TCP/IP) or the user datagram protocol (UDP).

In the proposed solution, one mosquito server [22] has been chosen as the MQTT *broker* deployed on an edge device. The mosquito MQTT broker can utilize two communication protocols, its the mqtt protocol based on TCP/IP and the websockets (ws) protocol. The websockets is a full-duplex communication protocol over a single TCP connection channel. The IETF standardized the websockets protocol as RFC 6455 in 2011 [23].

### The Firmware of *TactCube*

The ESP32 MCU embeds an Xtensa*®* dual-core 32-bit LX6 microprocessor, able to perform up to 600 MIPS. To develop the *TactCube* firmware, the open-source firmware NodeMCU [24] was loaded on its SoM. It was built on the Espressif*®* Non-OS Software Development Kit (SDK) platform offered by the producer of the ESP32 System on Chip (SoC). By deploying the NodeMCU on the *TactCube* device, it was possible to develop a Lua language-based, interactive firmware [25].

**Figure 9:**
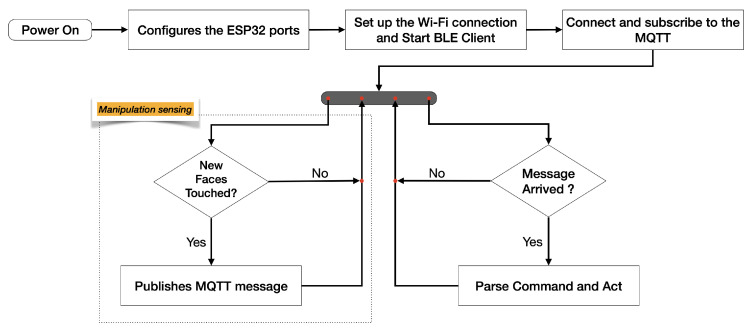
*TactCube* firmware workflow.

The NodeMCU programming model is asynchronous and event-driven. Then, without any real-time operating system (RTOS), it was possible to develop a firmware for the *TactCube* that communicates asynchronously and has real-time reactions to the user manipulations. Figure 9 shows a summarized workflow of the whole firmware.

In the following, the algorithm (Algorithm 1) of the manipulation sensing reported in Figure 9 is briefly described.
**Algorithm 1** Manipulation sensingOldTouchStatus←0**repeat**     **for all** $\left\{InputTouch_{i}|1\leq i\leq 5\right\}$  **do**         **if** $InputTouch_{i}$ is *Touched*  **then**             $InputTouch_{i}\leftarrow 1$         **else**             $InputTouch_{i}\leftarrow 0$         **end if**     **end for**    $\sum_{i=1}^{5}\left(Touched_{i}\leftarrow 2^{i}*InputTouch_{i}\right)$      ▹ (Equation (2)).     **if** TouchStatus≠OldTouchStatus  **then** Publish Message with TouchStatus        OldTouchStatus←TouchStatus     **end if****until** Deep Sleep Mode

A haptic feedback can be generated by publishing, on feedback *topic*, a message containing the pattern of the vibrations. Such a message must be a string containing integers separate by space characters so that the numbers in an odd position indicate the length in milliseconds of the vibration, while the numbers in an even position indicate the length in milliseconds of pause.

As an example, if the message payload sent contains: “500 500 1000 500 1000”, the result will be that: the motor vibrates 500 ms, then stops 500 ms and vibrates 1 s more, then stops 500 ms and vibrates 1 s more before stopping.

This solution gives broad flexibility in generating multiple feedback of arbitrary length.

## 5. Testing *TactCube* in Lab

To evaluate the behavior of the *TactCube* under test, a simple web application was developed. This tool allowed the operator performing the tests (namely *tester*) both to read the sequences of numbers the *TactCube* sends in real-time and to send the device some simple commands, such as "reset and calibrate", "vibration engine ON", and "vibration engine OFF". The tool has been developed using HTML5, CSS and JavaScript technologies. Since all these technologies are client side and can be executed on modern Web browser, it was unnecessary to deploy a Web server service on the edge device. A simple HTML page on the *tester*’s PC and the JavaScript Paho library [26], allowed us to subscribe and publish to the edge device MQTT broker by using the *websockets* protocol.

The *websockets* connection enables a two-way communication between a client running untrusted code in a browser and a remote host, which has opted-in to communications from that code, such as the Mosquito broker does in this case. This allows a web page to be updated with new data as soon as new messages are published on the broker, without a page reload or user action.

**Figure 10:**
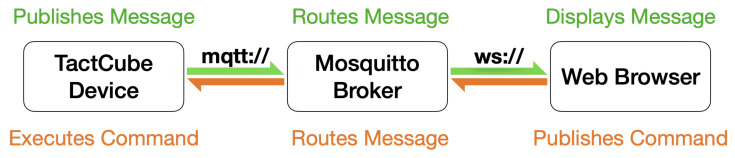
The two-way communications employed in the solution.

Figure 10 depicts the two-way communication solution with respective protocols used in the proposed solution.

To proceed with testing, the following infrastructure was set up at the LabGIS laboratory of the department of computer science at the University of Salerno:

- One NVIDIA*®* Jetson Nano™ Developer Kit board [27] with the Ubuntu 18.04.5 LTS OS, connected to the local area network (LAN) through an ethernet port, as edge device.
- One Wi-Fi access point connected to the LAN.
- Three BLE beacons placed in three place in the laboratory, to simulate three device with BLE advertising capability.
- One PC, connected to the LAN, for the tester, where the tester tool application was saved.

The procedure to test the *TactCube* device has been as follows. The *tester*’s PC and the *TactCube* device were connected to the same MQTT broker through the same Wi-Fi network. The *tester* opened the Web application tool and sent an initial reset command to the *TactCube*. The reset command initialized the touch sensors of the device. During this initialization, the *TactCube* must be left untouched to set the zero values of sensors. It is the offset to compare with values read during the manipulations to understand whether a face is touched or not. To signal the end of the calibration phase, the *TactCube* generated a brief vibration. After that, the *TactCube* can be used.

**Figure 11:**
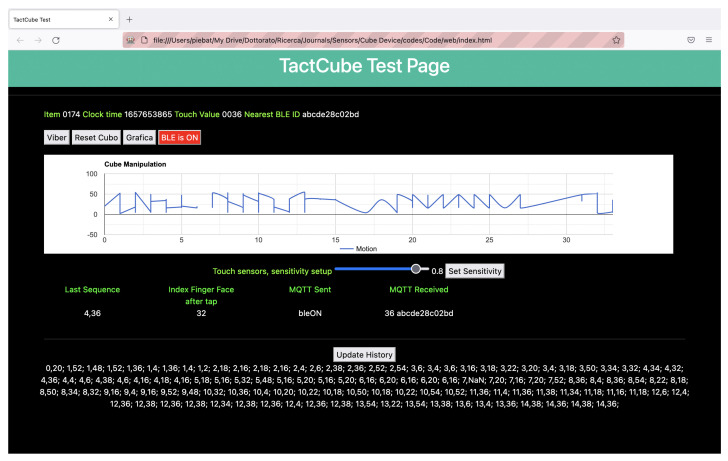
A screenshot from test tool.

After initialization, the *tester* grasped the *TactCube* in hand and started to randomly manipulate the *TactCube*, rotating and tapping on the faces under the index finger. The device sent a sequence of numbers according to Equation (3). Those numbers were visualized one by one on the screen in the textual form, while the sequences were displayed as a line graph, as Figure 11 shows. Next, the *tester* turned on the BLE feature of the *TactCube* and activated one BLE Beacon near it, verifying that the ’Nearest BLE ID’ was caught even while continuously rotating and tapping the *TactCube*.

## 6. Discussion

The experiment had the following goals:

- To validate the choices made for the construction of the device (components, programming environment, embodiment design);
- To validate the MQTT selection as the messaging communication protocol;
- To validate the use of touch sensors to define device rotation.

The experiment was repeated several times in the laboratory, and the authors’ considerations are given below.

The ESP32 Wi-Fi and BLE worked well even in coexistence. All the touches and the beacon identities were correctly visualized by the tester application.

The touch sensors demonstrated some issues related to the setup of their sensitivity. When the sensitivity was set too low, some steps of rotations were lost. When the sensitivity was set too high, some wrong data about the touched faces was detected. Indeed, with a high sensitivity, the *TactCube* acquired classified faces as touched that were not. Because the sensitivity of the tactile sensors required a combination of various parameters, many tests had to be conducted before an acceptable result was obtained. In this case, the use of the LUA development environment has been very appropriate. Indeed, it was possible to make changes to the firmware easily and quickly.

The MQTT solution was excellent in performance and implementation, with no problems or concerns raised about it.

The idea of using tactile sensors to read the rotation of the *TactCube* appeared satisfactory. The *TactCube* manipulation is quite different from performing gestures. The term gesture when discussing HCI commonly refers to hand or body gestures performed with respect to the plane of the ground and the direction of the force of gravity. In fact, many of the solutions proposed in the literature use accelerometers and gyroscopes [28]. The *TactCube* does not have these references; rather, it can be rotated by walking or waving the hand. Manipulations of the *TactCube* refer only to the three axes of the device itself; so its positioning relative to the floor or the user’s body [29] is not relevant. Moreover, avoiding the use of an accelerometer reduced costs in terms of components and power consumption.

**Figure 12:**
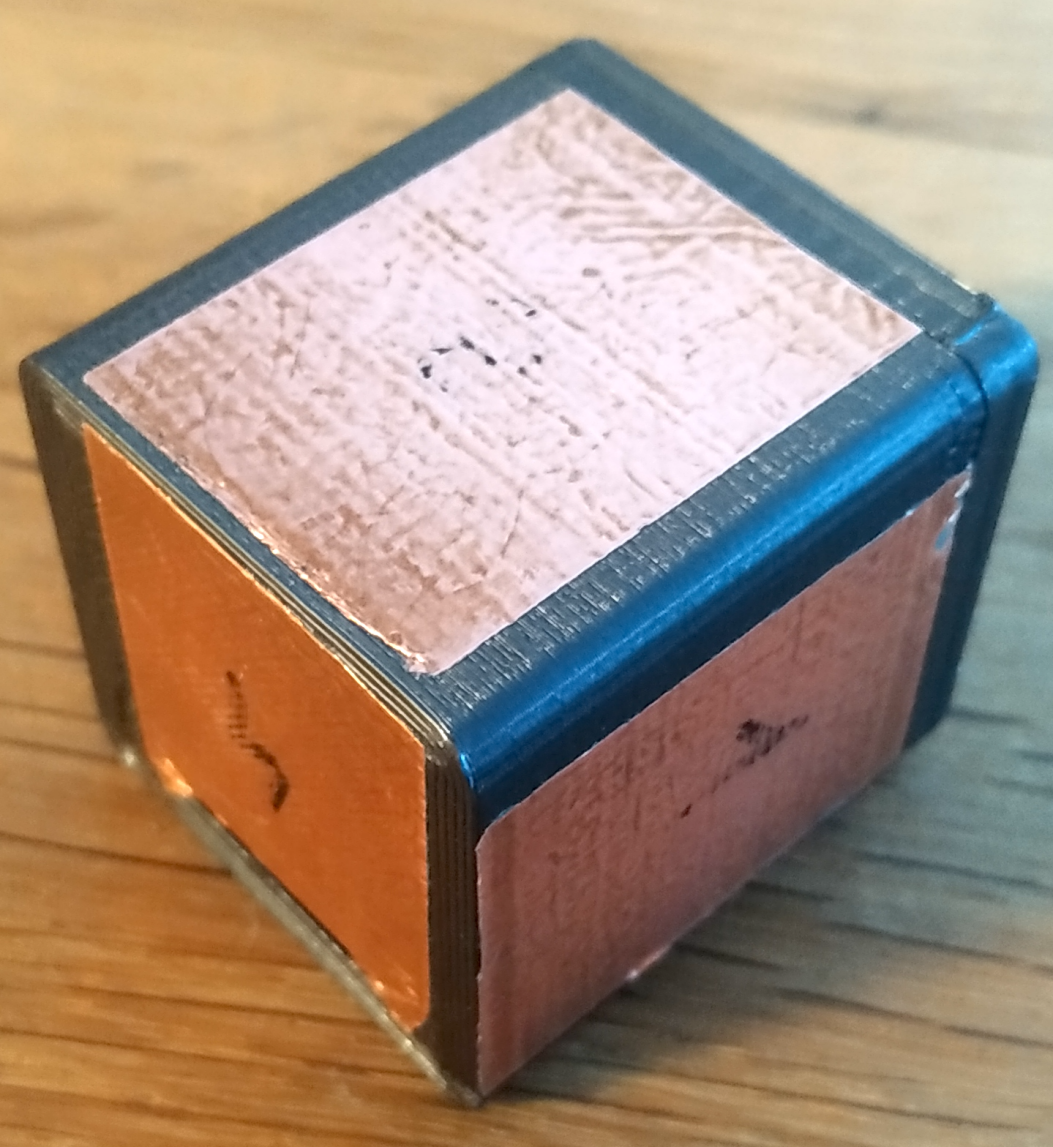
Smaller *TactCube* device for pocket sizes.

Although the design of the prototype allowed convenient manipulations by different-sized hands, insertion into a pocket was not as nimble. Thus, a new smaller pocket-sized device was made, eliminating the FIM module. Its dimensions of 38 mm × 34 mm × 34 mm enabled the desired results (Figure 12). The main difficulty in miniaturization has been met by battery size. Although a smaller device results in a handier device and better touch sensing, due to the shortening of circuit trace, a very small battery could require frequent charging, which could negatively affect the usability.

## 7. Conclusions

Manipulation of the *TactCube* generates sequences of numbers. Each number represents a precise position of the device in the user’s hand, detected by capacitive touch sensors. These sequences characterize a manipulation when associated with the sequence of numbers expressing the time intervals taken by the user to move from one position to another (Equation (5)). Although the possible rotations that the user can perform with the device are limited to turn forward, turn backward, turn left and turn right and their combinations, since the sequences can have different lengths and can be performed at different speeds, the set of possible manipulations can be virtually infinite.

The use of capacitive sensors for such a task has not been found in the literature. When compared with the more popular solution of using micro-electromechanical systems (MEMS), such as gyroscopes and accelerometers, the proposed solution is innovative. It offers the advantage of inexpensive components and low power consumption because it does not require external active components. In addition, MEMS components usually require sophisticated deep learning models just to recognize the patterns of interest (forward or backward rotation, etc.), and the results may still contain a large margin of uncertainty which must be mitigated by complicated calibration methods compared to the simple calibration used for the capacitive touch sensor [30].

There are countless learning tasks in the literature that require dealing with sequential data, such as time series prediction, video analysis, music information retrieval, language translation and handwriting recognition. In all these cases, a model must learn from inputs that are sequences [31], as in our case. Therefore, since long short-term memory (LSTM) and bidirectional recurrent neural network (BRNN) architectures have recently demonstrated revolutionary performance in language translation and handwriting recognition, it is believed that such artificial neural networks can be successfully investigated in future work to translate the data sequences generated by *TactCube* into sentences and messages that can be used to converse with the AmI.

Because the conversation is bidirectional, and the device can translate the sequence of data from the AmI into vibration sequences of varying length and frequency, users can learn to interpret this haptic feedback as messages from the AmI.

Furthermore, the *TactCube* achieves the basic requisite of a sensing-based interactive interface, indeed:

- The sensing-based interaction must be useful and active, but not disturb the user.
  - In fact, the touch-sensing actively detects how the *TactCube* is held in the hand, and the haptic vibration can gently communicate with users, without disturbing them or people nearby.
- The system must know that the user is addressing it and not other systems.
  - Because the *TactCube* connects with the AmI through a local area network and uses the MQTT protocol, listening to only the *topics* of interest and knowing the location of message sources dramatically reduces the possibility of misunderstandings.
- The user must know that the system is responding to his request.
  - With the envisioned solution, users have two ways to understand that the system is reacting to their actions: by interpreting haptic feedback and by observing the reactions of the AmI.
- The system must know which object the user’s command refers to.
  - The use of BLE is intended to discriminate neighboring devices by offering a solution to define the object to be commanded.

In conclusion, this paper has presented an innovative idea to converse with the AmI using only the tactile sense, proposing an innovative device that can be inexpensive and easy to produce.

### Future Work

A more accurate usability study involving several users is left as future work along with the creation of a labeled dataset suitable to pre-train a BRNN model.

## Figures and Tables

**Table 1:** Capacitance composition.

$C_{ground}$	Capacitance between the circuit ground and the earth
$C_{component}$	ESP32’s intrinsic parasitic capacitance
$C_{trace}$	Parasitic capacitance between the trace and the circuit ground
$C_{electrode}$	Parasitic capacitance between the touch electrode and the circuit ground
$C_{touch}$	Capacitance formed by a finger and the touch electrode relative to the earth

## Abbreviations

The following abbreviations are used in this manuscript:

AmI	Ambient Intelligence
AI	Artificial Intelligence
HCI	Human-Computer Interaction
TUI	Tangible User Interface
GUI	Graphic User Interface
NN	Neural Network
SoM	System on Module
MCU	Microcontroller Unit
LiPo	Lithium Polymer Batery
FIM	Fingerprint Identification Module
TTL	Tramsistor-Transistor Logic
VM	Vibrating Motor
GPIO	General Purpose Input Output
ULP	Ultra Low Power
BLE	Bluetooth Low Energy
RSSI	Received Signal Strength Indicator
IP	Internet Protocol
TCP	Transmission Control Protocol
UDP	User Datagram Protocol
ws	Websockets
SDK	Software Development Kit
SoC	System on Chip
RTOS	Real-Time Operating System
LAN	Local Area Network
LSTM	Long-Short Term Memory
BRNN	Bidirectional Recurrent Neural Networks
